# Recurrent Sleep Fragmentation Induces Insulin and Neuroprotective Mechanisms in Middle-Aged Flies

**DOI:** 10.3389/fnagi.2016.00180

**Published:** 2016-08-02

**Authors:** Michael J. Williams, Emelie Perland, Mikaela M. Eriksson, Josef Carlsson, Daniel Erlandsson, Loora Laan, Tabusi Mahebali, Ella Potter, Robert Frediksson, Christian Benedict, Helgi B. Schiöth

**Affiliations:** Functional Pharmacology, Department of Neuroscience, Uppsala UniversityUppsala, Sweden

**Keywords:** sleep, metabolism, insulin, glucagon, dopamine, molecular chaperone, *Nrf2*

## Abstract

Lack of quality sleep increases central nervous system oxidative stress and impairs removal of neurotoxic soluble metabolites from brain parenchyma. During aging poor sleep quality, caused by sleep fragmentation, increases central nervous system cellular stress. Currently, it is not known how organisms offset age-related cytotoxic metabolite increases in order to safeguard neuronal survival. Furthermore, it is not understood how age and sleep fragmentation interact to affect oxidative stress protection pathways. We demonstrate sleep fragmentation increases systems that protect against oxidative damage and neuroprotective endoplasmic reticulum molecular chaperones, as well as neuronal insulin and dopaminergic expression in middle-aged *Drosophila* males. Interestingly, even after sleep recovery the expression of these genes was still upregulated in middle-aged flies. Finally, sleep fragmentation generates higher levels of reactive oxygen species (ROS) in middle-aged flies and after sleep recovery these levels remain significantly higher than in young flies. The fact that neuroprotective pathways remain upregulated in middle-aged flies beyond sleep fragmentation suggests it might represent a strong stressor for the brain during later life.

## Introduction

Sleep fragmentation, characterized by recurrent awakenings after sleep onset, not only affects the elderly, those who suffer from sleep apnea and bipolar patients (Carskadon et al., 1982; Reutrakul and Van Cauter, 2014), but also shift workers (Reynolds and Banks, 2010). One current hypothesis maintains that sleep allows for the removal of neurotoxic byproducts that accumulate in the central nervous system during the awake period (Xie et al., 2013). During sleep, it is possible that toxic metabolites are removed from the brain by convective flow between the cerebrospinal and interstitial fluid, possibly to reduce neuronal exposure to factors that are neurodegenerative. This was evidenced by the removal of tracer from the mouse brain during sleep, however, this removal was diminished by ~95% when sleeping animals were awakened from sleep (Xie et al., 2013). This indicates that consolidated sleep may be especially critical for ‘cleaning up’ the interstitial space of the brain. What is not understood is how increases in fragmented sleep – e.g., as it occurs during aging -, alter expression and efficiency of cytoprotective systems that promote neuronal survival.

It is postulated that lack of sleep quality produces an oxidative challenge for the brain and that sleep has a protective role against oxidative damage. It must be stated that this is controversial and there is evidence for and against the idea of an interaction between oxidative stress and sleep (Ramanathan et al., 2002; Gopalakrishnan et al., 2004; Koh et al., 2006). In *Drosophila* it was shown that inducing oxidative stress increases sleep fragmentation as flies age (Koh et al., 2006). Furthermore, in rats it was published that sleep deprivation decreases the expression of certain antioxidant enzymes (Ramanathan et al., 2002). However, in rats, even after what could be considered long-term sleep deprivation (14 days) no obvious neuronal damage due to oxidative stress was observed (Gopalakrishnan et al., 2004). It is speculated that some regions of the brain may be more prone to oxidative stress than others, such as dopaminergic-rich regions (Cirelli, 2006; Fu et al., 2016).

To understand if sleep fragmentation can enhance neuronal degeneration we studied the expression of genes known to be regulated by oxidative stress or neuronal damage. One mechanism by which cells defend against oxidative injury is through increased transcription of antioxidant response element (ARE) genes. ARE is a *cis*-acting enhancer sequence that regulates many cytoprotective genes via the transcription factor nuclear factor, erythroid 2-like 2 (NFE2L2; Johnson et al., 2008). Of note, in mice *Nfe2l2* was upregulated after just 6 h of sleep deprivation (Nikonova et al., 2010). Yet, it was demonstrated, *in vitro*, that Nfe2l2-dependent transcription can prevent reactive oxygen species (ROS)-induced apoptosis in neurons and astrocytes (Johnson et al., 2008). Also, expression of ARE-driven genes, such as *NAD(P)H dehydrogenase, quinone 1* (*NQO1*) and *heme oxygenase (decycling) 1* (*HO-1*), is increased in brain tissue in a Parkinson’s disease (PD) mouse model (Jing et al., 2015), which could be a neuroprotective response mediated by NFE2L2 activation.

Although it is not fully understood how oxidative stress causes cellular degeneration, it may induce mitochondrial damage, leading to reduced levels of ATP, which in turn activates the endoplasmic reticulum (ER) unfolded protein response (UPR; Cano et al., 2014). Interestingly, in mice and fruit flies, a decrease in sleep quantity activates the UPR (Shaw et al., 2000; Naidoo et al., 2005, 2007). Furthermore, recent evidence in *Drosophila* indicates that lowering levels of ER stress improves age-related decreases in sleep consolidation (Brown et al., 2014). However, extended ER stress and upregulation of the UPR induces autophagy, leading to apoptosis (Senft and Ronai, 2015).

Insulin signaling may protect against UPR induced apoptosis by inhibiting cytochrome c release from mitochondria (Sanderson et al., 2013). Furthermore, in a mouse model of Alzheimer’s disorder (AD), slow-release of IGF-1 systemically enhanced cognitive performance, decreased amyloid levels and protected synapses (Butovsky et al., 2006). Moreover, in mouse models of AD, insulin and IGF-1 signaling was found to be impaired, and increasing their central nervous system levels was neuroprotective (Freiherr et al., 2013). However, in *Drosophila* it was reported that decreasing systemic insulin signaling throughout adult life lowers the incidence of sleep fragmentation in elderly flies, most likely by slowing the aging process (Metaxakis et al., 2014). On the other hand, another report demonstrated that upregulating insulin signaling in *Drosophila* actually increased sleep quality (Cong et al., 2015). This illustrates that the benefits and effects of insulin signaling when it comes to sleep are still not understood, especially when it comes to age-related sleep disruptions.

If quality sleep is necessary for the clearance of toxic biomolecules we should expect significant correlations between the amount and/or quality of sleep and human neurodegenerative diseases. Interestingly, poor sleep quality, especially among the elderly, is linked to an increased risk of PD and AD (Benedict et al., 2014; Schrempf et al., 2014). Yet, why sleep disturbance is associated with aging and aging-related disease is still not clear. Ours is the first study to examine important concomitant neuroprotective systems, comparing how young and middle-aged flies respond to recurrent sleep fragmentation. We show that sleep fragmentation induces conserved neuroprotective systems in young and middle-aged flies. Moreover, middle-aged flies not only induce multiple protective systems, but still maintain higher levels of expression after they are allowed to recover from disturbed sleep. Finally, we show that ROS concentrations in the brains of sleep-fragmented middle-aged flies are higher than in young flies and that this increase remains after sleep recovery.

## Materials and Methods

### Fly Stocks and Maintenance

The *CSORC* lab wild-type strain was created by outcrossing *Canton S* and *Oregon R-C* flies [both strains were received from the Bloomington Stock Center^1^)], for 10 generations. All flies, unless otherwise stated, were maintained on enriched Jazz mix standard fly food (Fisher Scientific). Flies were maintained at 25°C in an incubator at 60% humidity on a 12:12 light:dark cycle.

### RNA Purification

The phenol-chloroform method was used for RNA extraction from tissue samples (Chomczynski and Sacchi, 1987). 25 fly heads or 10 whole flies were homogenized with 800 μl TRIzol (Invitrogen, USA), 200 μl Chloroform (Sigma–Aldrich) was added and samples were centrifuged at 12000 rpm for 15 min at 4°C. The aqueous layer, which contained RNA, was separated and 500 μl isopropanol (Solvaco AB, Sweden) was added. The RNA was precipitated by storing the samples at -32°C for 2 h. Samples were centrifuged at 12000 rpm for 10 min at 4°C, to collect the RNA pellets, which were then washed with 75% ethanol (Solvaco AB, Sweden) to remove the organic impurities. Samples were allowed to air dry to remove any traces of ethanol. Dried RNA pellets were dissolved in 21.4 μl of RNAse free water (Qiagen GmBH, Germany) and 2.6 μl of DNAse incubation buffer (Roche GmBH, Germany). The samples were incubated at 75°C for 15 min to ensure complete dissolution of RNA-pellets. 2 μl of DNAse I (10 U/μl, Roche GmBH, Germany) was added to each sample, and incubated at 37°C for 3 h to remove DNA contamination. DNAse was deactivated by incubating the samples at 75°C for 15 min. Removal of DNA was confirmed by PCR using Taq polymerase (5 U/μl, Biotools B & M Labs, Spain), followed by agarose gel electrophoresis. The RNA concentration was measured using a nanodrop ND 1000 spectrophotometer (Saveen Werner).

### cDNA Synthesis

cDNA was synthesized from RNA template using dNTP 20 mM (Fermentas Life Science), random hexamer primers and M-MLV Reverse Transcriptase (200 U/μl, Invitrogen, USA) by following manufactures instructions. cDNA synthesis was confirmed by PCR followed by agarose gel electrophoresis.

### quantitative RT-PCR (qRT-PCR)

Relative expression levels of three housekeeping genes (*EF-1, Rp49*, and *RpL11*) and of the genes of interest were determined with quantitative RT-PCR (qPCR). Each reaction, with a total volume of 20 μl, contained 20 mM Tris/HCl pH 9.0, 50 mM KCl, 4 mM MgCl_2_, 0.2 mM dNTP, DMSO (1:20), and SYBR Green (1:50000). Template concentration was 5 ng/μl and the concentration of each primer was 2 pmol/μl. Primers were designed with Beacon Designer (Premier Biosoft) using the SYBR Green settings. All qPCR experiments were performed in duplicates; for each primer pair a negative control with water and a positive control with 5 ng/μl of genomic DNA were included on each plate. Amplifications were performed with 0.02 μg/ml Taq DNA polymerase (Biotools, Sweden) under the following conditions: initial denaturation at 95°C for 3 min, 50 cycles of denaturing at 95°C for 15 s, annealing at 52.8–60.1°C for 15 s and extension at 72°C for 30 s. Analysis of qPCR data was performed using MyIQ 1.0 software (Bio-Rad) as previously reported (Lindblom et al., 2006). Primer efficiencies were calculated using LinRegPCR (Ramakers et al., 2003) and samples were corrected for differences in primer efficiencies. The GeNorm protocol described by Vandesompele et al. (2002) was used to calculate normalization factors from the expression levels of the housekeeping genes. Grubbs’ test was performed to remove outliers. Differences in gene expression between groups were analyzed with ANOVA followed by Fisher’s PLSD test where appropriate. *P* < 0.05 was used as the criterion of statistical significance. The following primers were used: *EF-1* F: 5′-GCGTGGGTTTGTGATCAGTT-3′, R: 5′-GATCTTCTCCTTGCCCATCC-3′; *Rp49* F: 5′-CACACCAAATCTTACAAAATGTGTGA-3′, R: 5′-AATCCGGCCTTGCACATG-3′; *RpL11* F: 5′-CCATCGGTATCTATGGTCTGGA-3′, R: 5′-CATCGTATTTCTGCTGGAACCA-3′; *Aps* F: 5′-GAGAACCAACCATACACAT-3′, R: 5′-CATAAATGAACGCCTTGAC-3′; *Keap1* F: 5′-GGTGGTCGATTCGGATCGG-3′, R: 5′-TGGCGTATTTGGACATGCAGA-3′; *cnc* F: 5′- GAATGACCGCCGATCTCTTGG-3′, R: 5′- GGAGCCCATCGAACTGACA-3′; *CaBP1* F: 5′- GCAGCGTTAGTGCCTTCTATT-3′, R: 5′- CTTTCAGCACCTCCCGGTC-3′; *ERp60* F: 5′- GACTTTGCCACCACCCTAAAA-3′, R: 5′- TACTCGGGCTTCAATCGCTTG-3′; *Crc* F: 5′- GAAAACTGGGAGGATACGTGG-3′, R: 5′- GAGAGGTCTGAATGCCTTTGTC-3′; *ple* F: 5′–ATCAAGAAATCCTACAGTAT-3′, R: 5′-CACAATGCAATCTTCCAG-3′; *Vmat* F: 5′-CGTGACCTTCGGGACGATAG-3′, R: 5′-ACTAGAGCGGGAAAACCAGC-3′; *DAT* F: 5′-GCTTCAAACCATAAGTTCTAA-3′, R: 5′-TCGGACTTGATATTATCTACAA-3′; *Ilp2* F: 5′-TCTGCAGTGAAAAGCTCAACGA-3′, R: 5′-TCGGCACCGGGCATG-3′; *Ilp3* F: 5′-TGAACCGAACTATCACTCAACAGTCT-3′, R: 5′-AGAGAACTTTGGACCCCGTGAA-3′; *Ilp5* F: 5′-GAGGCACCTTGGGCCTATTC-3′, R: 5′-CATGTGGTGAGATTCGGAGCTA-3′; *Ilp6* F: 5′-GTCCAAAGTCCTGCTAGTCCT-3′, R: 5′-TCTGTTCGTATTCCGTGGGTG-3′; *Akh* F: 5′-CTGGTCCTGGAACCTTTT-3′, R: 5′-GAGCTGTGCCTGAGATTG-3′; *InR* F: 5′-CCAAGAAGTTCGTCCATC-3′, R: 5′-TCATTCCAAAGTCACCAA-3′.

### Locomotion, Sleep Fragmentation, and Sleep Behavior

For locomotion and sleep analysis, newly enclosed flies were collected and raised at 25°C, 60% humidity, on a 12:12 light:dark cycle for either 5–7 or 25–27 days. Sleep fragmentation was achieved by turning on the lights once an hour for 30 min during the 12 h dark cycle. This was repeated for a total of four nights. These were considered sleep fragmented flies. Another group of flies were exposed to the same sleep fragmentation regime but afterward were allowed to recover for 4 days on a normal 12:12 h day:night cycle. This was the sleep fragmented plus recovery group. The same light was employed for both the 12 h light cycle and sleep fragmentation during the dark cycle. The light used was a full spectrum bulb at 600 lux. Locomotor activity was collected using the *Drosophila* activity monitoring system (DAMS; Trikinetics) for 4 days at 25°C, 60% humidity, on a 12:12 light:dark cycle. Locomotor activity was collected in 1 min bins and analyzed with PySolo (Gilestro and Cirelli, 2009). Total sleep, average sleep length and average sleep bouts was calculated based on the standard sleep definition as a period of 5 or more minutes of inactivity. Flies were allowed to adapt to the DAMS system for 24 h before analysis was performed. The assay was repeated at least three times with 30 males used for each genotype (*N* = 90).

### Reactive Oxygen Species (ROS) Detection

To detect the relative level of ROS in the heads a similar protocol was followed (Owusu-Ansah and Banerjee, 2009; Wang et al., 2011). Briefly, heads of young (5–7 days) and middle-aged (25–27 days) flies (three per assay) were homogenized in 15 μl of cold lysis buffer pH 7.4 (50 mM HEPES, pH 7.4, 5 mM CHAPS, and 5 mM DTT). The solutions were transferred to a 96-well plate and 20 μl of dihydroethidium (DHE, Cayman Chemical) was added to the head homogenate to a final concentration of 10 μM. The reaction mixture was incubated at 25°C for 10 min in the dark. The fluorescence intensity was measured at 405 nm on a fluorescence microplate reader (LabSystems Multiskan MS). The results were analyzed using Ascent 2.6 software. At least seven replicates were run in triplicate for each experimental condition.

### Fluorescence Microscopy

Fluorescence microscopy (Axioplan 2 imaging, Zeiss) was used to identify GFP expressing dopaminergic neurons in whole brains from the F1 generation of *Drosophila* males. The Zeiss AxioVision computer software was used to conduct z-stack imaging of the GFP labeled dopaminergic neurons. Z-stack imaging was performed by choosing “Multidimensional acquisition/Z” and defining start and end points for the z-stack. The number of slices chosen for z-stack images varied between 37 and 38 photos. Slice thickness was set to an optimal distance of 3.850 μm. Only one green fluorescence filter, (Fluorescein FITC, Ex: 494, Em: 517) was used to detect GFP labeled dopaminergic neurons. The Alexa Fluor (488 nm, Ex: 499, Em: 520) dye was selected in the “Multidimensional Acquisition/C” for correct calculation of the optimal distance. The “Live” view was used to enable real time video and check for under- and overexposure. Overexposed pixels were shown as red dots and underexposed pixels were shown as blue dots. Corrections of under- and overexposure was made by slowly rotate the focus knob until the image became clear and sharp. Exposure time was set to 824 ms (Mode: fixed), the same exposure time was used for all photos. Single images were saved in the ZVI format (Carl Zeiss) to ensure complete storage of all metadata. The ImageJ software was used for analysis of images obtained from the fluorescence microscopy. A total number of 20 z-stack images of different brains for each group were included in the analysis. In the ImageJ software, sum slices projections (containing the sum of the slices in the stack) were created from all z-stack images and image rotation was performed for optimal positioning of the fly brain. The GFP expression of dopaminergic neurons was estimated by fluorescence intensity quantification in the region of interest (ROI manager) including sleep regulating structures (mushroom bodies and pars intercerebralis). Background intensity was measured for each image in an area adjacent to the region of interest. Mean background intensity was subtracted from the mean fluorescence intensity in the region of interest. Total mean fluorescence intensity was calculated for each group and used for statistical analysis.

### Statistical Analysis

Mean and standard error from all replicates of each experiment was calculated. All analysis was performed with GraphPad Prism 4, and used ANOVA with appropriate *post hoc* analysis for multiple comparisons. The type of analysis performed for each assay is specified in the appropriate figure legend.

## Results

### Sleep Fragmentation Profile in Young and Old Flies

**FIGURE 1:**
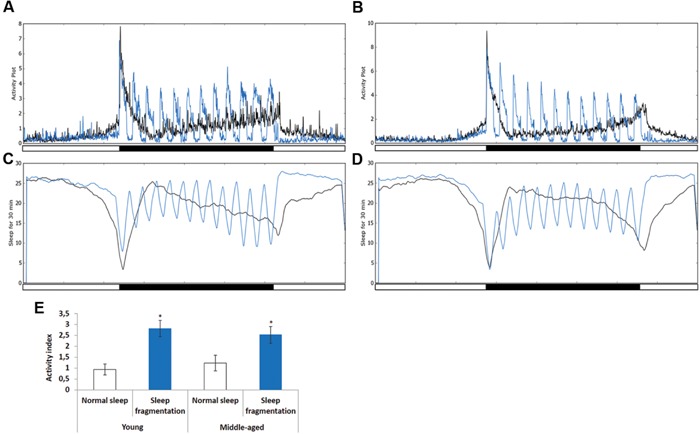
**Sleep fragmentation induces rebound sleep. (A,C)** Averaged locomotor activity over a 24 h period of **(A)** 5–7 days old or **(B)** 25–27 days old males kept on either a 12:12 light dark cycle (gray line) or sleep fragmented (purple line). **(C,D)** Averaged sleep during a 30 min period of 24 h. **(C)** 5–7 days old or **(D)** 25–27 days old males on either a 12:12 light dark cycle (gray line) or sleep fragmented (purple line; *n* = 90). **(E)** Average activity comparing 30 min light-stimulus phase of sleep fragmented males with the same time periods for males kept on a 12:12 light dark cycle (*n* = 90; *P < 0.05, compared with controls, one-way ANOVA with Tukey’s *post hoc* test for multiple comparisons). Error bars indicate SEM.

To investigate the effects of sleep fragmentation on young (5–7 days old) and middle-aged (25–27 days old) *Drosophila* males, flies were subjected to 30 min of light every hour during the dark cycle (**Figures 1A–D**). In both young and middle-aged flies this light-stimulus induced activity (**Figures 1A–D**, blue line). In fact, on average there was a threefold increase in activity during the 30 min light-stimulus phases in both young and middle-aged flies (**Figure 1E**). Interestingly, during the interim 30 min flies had increased sleep compared to those on a normal sleep/wake cycle (**Figures 1B,D**). Furthermore, after the flies were sleep fragmented a significant sleep rebound was observed during the day (**Figures 1B,D**).

**FIGURE 2:**
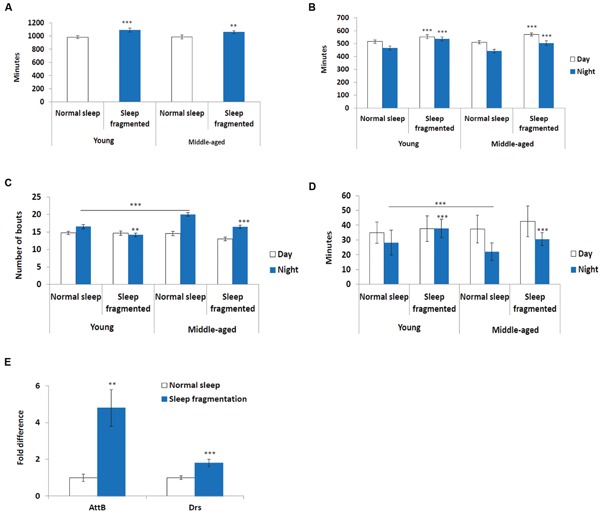
**Sleep fragmented flies sleep more than normally maintained flies. (A–D)** Analysis of sleep behavior comparing flies on a normal 12:12 light:dark cycle with flies undergoing sleep fragmentation. **(A)** Total sleep during a 24 h period. **(B)** Average number of minutes flies slept during the 12 h light or dark period. **(C)** Average number of sleep bouts during the 12 h light or dark period. **(D)** Average sleep episode length during the 12 h light or dark period. (**A–D**: *n* = 60 flies. **P < 0.01, ***P < 0.005 compared with controls, A non-parametric Kruskal–Wallis test was performed). **(E)** qPCR was performed to determine if sleep fragmentation was able to induce an inflammatory response. Both AttacinB (AttB) and Drosomycin (Drs) antimicrobial genes where induced by sleep fragmentation. (All assays were repeated at least seven times. *n* = 25 male heads per treatment. **P < 0.01, ***P < 0.005 compared with controls, one-way ANOVA with Tukey’s *post hoc* test for multiple comparisons). In all graphs error bars indicate SEM.

Next, we used the pySolo program (Gilestro and Cirelli, 2009) to carefully examine the effect sleep fragmentation had on young versus middle-aged males. Surprisingly, over a 24 h period sleep fragmented flies, both young (*P* < 0.005) and middle-aged (*P* < 0.01), slept significantly more than flies on a normal sleep/wake cycle (**Figure 2A**). In both young and old flies the increase in total sleep was split between day (*P* < 0.005) and night (*P* < 0.005) (**Figure 2B**). These results demonstrated that our sleep fragmentation intervention did not reduce sleep quantity.

Previously, it was reported that old flies (55 days) suffer from fragmented sleep (Metaxakis et al., 2014), and we observed that this was true only during the night in middle-aged flies, where middle-aged males had significantly more and shorter sleep bouts compared to young males (*P* < 0.005; **Figures 2C,D**). Interestingly, sleep fragmentation significantly reduced the number of nighttime sleep bouts in young flies (*P* < 0.01; **Figure 2C**). Furthermore, sleep fragmentation increased the number of minutes each fly slept per bout at night in all males (*P* < 0.005; **Figure 2D**). This indicates that during the interim where the lights were off the sleep fragmented flies had nearly uninterrupted sleep (**Figures 1A–D**).

Similar to humans, sleep restriction induces an inflammatory response in *Drosophila* (Williams et al., 2007). We performed qPCR and measured the transcript levels of two antimicrobial genes known to be induced in sleep restricted flies, *Attacin-B* (*AttB*) and *Drosomycin* (*Drs*), to understand if our sleep intervention induced an inflammatory response (Williams et al., 2007). In our sleep fragmented males both of these genes were significantly induced (*AttB P* < 0.01; *Drs P* < 0.005; **Figure 2E**).

### Sleep Fragmentation Induces *Nrf2* Expression

**FIGURE 3:**
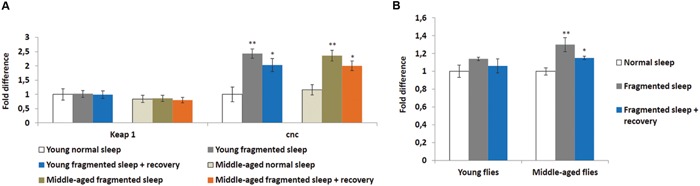
**Sleep fragmentation induces the oxidative stress pathway. (A,B)** Either 5–7 or 25–27 days old control, sleep fragmented or males allowed to recover for 4 days after sleep fragmentation were used. **(A)** The expression of the cellular stress regulating *KEAP1-NFE2L2* system was assessed. In *Drosophila NFE2L2* is known as *cap-n-collar* (*cnc*). **(B)** Reactive oxygen species (ROS) levels in young and middle aged males. All assays were repeated at least seven times. (**A**: *n* = 25 male heads per treatment. **B**: *n* = 3 male heads per treatment; *P < 0.05, **P < 0.01 compared with controls, one-way ANOVA with Tukey’s *post hoc* test for multiple comparisons). In all graphs error bars indicate SEM.

Sleep deprivation was shown to induce oxidative stress and the KEAP1/NFE2L2 pathway is a major regulator of cytoprotective responses to endo- and exogenous stresses caused by ROS (Cheng et al., 2011). Therefore, we performed qPCR to look at the head expression levels of the *Drosophila KEAP1* and *NFE2L2* homologs, *Keap1* and *cap-n-collar* (*cnc*), respectively. Sleep fragmentation of young or middle-aged flies had no significant effect on the expression of *Keap1*, while that of *cnc* was significantly induced in the heads of all males (sleep fragmented *P* < 0.01; sleep fragmented + recovery *P* < 0.05; **Figure 3A**). Thus, even though sleep fragmented flies sleep more than controls, similar to sleep deprivation, fragmentation induced a cytoprotective pathway typically activated during times of oxidative stress.

To establish if sleep fragmentation had an effect on ROS production in the brain, ROS levels were measured in heads from young and middle-aged males. First, age had no significant effect on ROS levels when normal slept flies were compared (**Figure 3B**). Yet, ROS levels were significantly elevated in sleep fragmented middle-aged males (*P* < 0.01), whereas there was only a small increase in young flies (**Figure 3B**). Furthermore, even after 4 days of recovery sleep, ROS levels were still significantly elevated in the brains of older males (*P* < 0.05; **Figure 3B**).

### Sleep Fragmentation Induces ER-Chaperones

**FIGURE 4:**
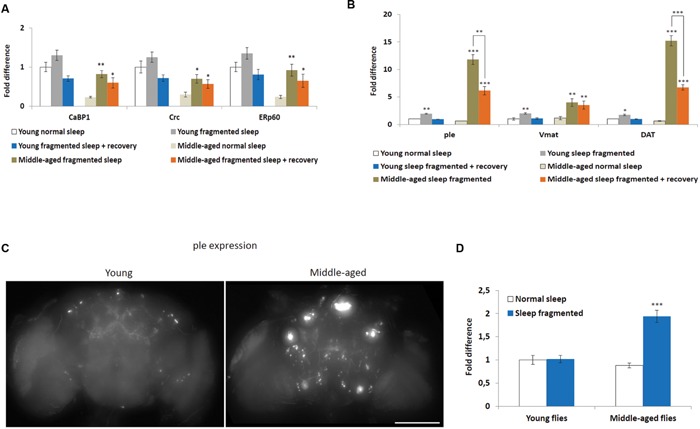
**Sleep fragmentation induces ER molecular chaperone and dopaminergic pathways. (A–D)** Either 5–7 or 25–27 days old control, sleep fragmented or males allowed to recover for 4 days after sleep fragmentation were used. **(A)** The expression of endoplasmic reticulum molecular chaperones *CaBP1*, *Crc*, and *ERp60* was assessed. **(B)** The expression of genes involved in dopamine production or signaling (*ple*, *Vmat*, and *DAT)* were assessed. (**A,B**: *n* = 25 male heads per treatment, *P < 0.05, **P < 0.01, ***P < 0.005 compared with controls, one-way ANOVA with Tukey’s *post hoc* test for multiple comparisons). **(C)** Brains from with young or middle-aged *ple-GAL4;UAS-GFP* males that were sleep fragmented for 4 days. Fluorescence indicates *ple* transcription in dopaminergic neurons. Presented as a Z-project (*Z* = 2 μm, 75 slices total Size bar = 200 μM). **(D)** Mean brain fluorescence intensity between young and middle-aged normal sleep and sleep fragmented groups. Normal slept young flies were set as 100%, represented as 1 on the graph. (**D**: *n* = 20 male heads per group, ***P < 0.05 compared with controls, one-way ANOVA with Tukey’s *post hoc* test for multiple comparisons). In all graphs error bars indicate SEM.

A number of cellular stresses, including oxidative stress, induce protein misfolding in the ER, causing ER stress, which, if prolonged, can lead to apoptosis (Han et al., 2013; Weids et al., 2016). However, moderate ER stress is cytoprotective and cells respond in various ways, including transcriptional activation of ER-chaperones, such as *PDIA6* (*Drosophila Calcium-binding protein 1, CaBP1*), *PDIA3* (*Drosophila ERp60*), and *Calreticulin* (*CALR*, *Drosophila Calreticulin, Crc*) (Sano and Reed, 2013). In fact, ER-chaperones are specifically linked with the neuroprotection of dopaminergic neurons in mice and rats (Dukes et al., 2008; Lessner et al., 2010). We performed qPCR to look at expression levels of *CaBP1*, *ERp60*, and *Crc* in heads of flies either maintained on a normal sleep schedule, after sleep fragmentation or after sleep fragmentation plus 4 days recovery. First, all ER-chaperone genes tested were expressed at significantly lower levels in normal slept middle-aged males, when compared to young males (*P* < 0.01; **Figure 4A**). Secondly, in middle-aged males sleep fragmentation induced a significant increase in ER-chaperone gene expression (*CaBP1 P* < 0.01, *Crc P* < 0.05, *ERp60 P* < 0.01) that was still elevated after recovery sleep (*P* < 0.05). Again, this indicates that older flies may be more susceptible to neuronal stress. It also indicates that they need to maintain multiple neuroprotective pathways as their brain might be more susceptible to neurodegenerative effects of sleep fragmentation.

Given that there is evidence the ER molecular chaperon complex may help in dopaminergic neuron survival (Lee et al., 2003; Lessner et al., 2010), we determined if sleep fragmentation influenced dopaminergic signaling. To do this qPCR was performed to look at three genes known to be involved in dopamine production, secretion or reuptake, pale (*ple*, *Drosophila tyrosine hydroxylase*), *Vesicular monoamine transporter* (*Vmat*), and *Dopamine transporter* (*DAT*). Interestingly, in young and middle-aged males, sleep fragmentation upregulated the expression of all genes. Yet, their expression was significantly more induced in middle-aged males (young males: *ple* and *Vmat P* < 0.01, *DAT P* < 0.05; middle-aged males *ple* and *DAT P* < 0.005, *Vmat p* < 0.01; **Figure 4B**). Furthermore, in middle aged males the expression remained elevated after 4 days recovery sleep (males *ple* and *DAT P* < 0.005, *Vmat p* < 0.01), while in young males expression levels returned to normal (**Figure 4B**).

To confirm that sleep fragmentation had a stronger effect on dopaminergic signaling in middle-aged flies, we crossed *ple-GAL4* driver flies with *UAS-GFP* expression flies and observed GFP expression in the *F*_1_ generation. If *ple* transcription was increased this would lead to increased GFP levels. When we compared levels after sleep fragmentation it was obvious there was more GFP expression in the brains of middle-aged flies (**Figure 4C**). By measuring GFP expression we determined that middle-aged sleep fragmented males expressed significantly more GFP, and thus *ple*, than all other groups (*P* < 0.005; **Figure 4D**). From this we conclude that sleep fragmentation significantly increases dopamine production in middle-aged males, most likely leading to increased dopaminergic signaling.

### Sleep Fragmentation Induces Insulin-Like Peptides and Insulin Receptor in Older Flies

**FIGURE 5:**
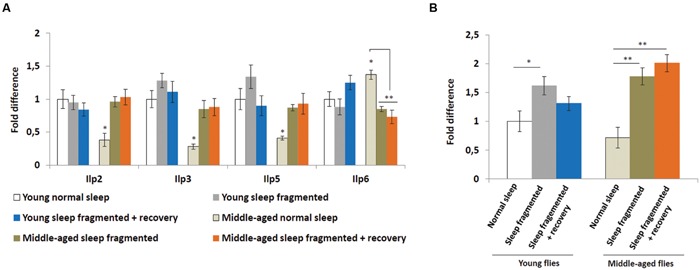
**Sleep fragmentation induces the insulin pathway. (A)** Relative expression levels of insulin-like peptides (Ilps). **(B)** Relative expression level of *Insulin-like receptor* (*InR*). **(A,B)** All assays were repeated at least seven times. Error bars indicate SEM. (For all genes, except *Ilp6*, *n* = 25 male heads per treatment; for *Ilp6*, *n* = 10 male whole bodies per treatment; *P < 0.05, **P < 0.01 compared with controls, one-way ANOVA with Tukey’s *post hoc* test for multiple comparisons).

Insulin signaling is also hypothesized to be neuroprotective (Freiherr et al., 2013) and it was discovered that manipulating insulin-like peptide expression influenced sleep fragmentation in elderly flies (Metaxakis et al., 2014), consequently we performed qPCR analysis on adult males to examine the expression of *Drosophila* insulin-like peptides, as well as the insulin receptor (*InR*). In *Drosophila*, a subset of median neurosecretory cells (MNCs), known as the insulin producing cells (IPCs), located in the brain produce insulin-like peptides (Ilp2, Ilp3, and Ilp5) associated with glucose metabolism (Broughton et al., 2005). Another insulin-like peptide, Ilp6, is produced by the fat body and signals back to the IPCs to inhibit *Ilp2*, *Ilp3*, and *Ilp5* expression (Bai et al., 2012). As reported previously (Metaxakis et al., 2014), when compared to young males, the heads of older males had significantly lower levels of *Ilp2*, *Ilp3*, and *Ilp5* (*P* < 0.05; **Figure 5A**). Since the IPCs produced Ilps signal to the fat body to inhibit dFoxo activity, which is necessary for *Ilp6* transcription, it was not surprising that the bodies of middle-aged flies had significantly higher levels of *Ilp6* (*P* < 0.05; **Figure 5A**). We examined *Ilp* expression immediately after sleep fragmentation, as well as after 4 days of recovery (**Figure 5A**). Sleep fragmentation had little effect on *Ilp* transcription in young males. However, in sleep fragmented middle-aged males, *Ilp2*, *Ilp3*, and *Ilp5* expression was induced to the same levels as normal slept young males; 4 days of recovery did not reduce their expression (**Figure 5A**). Furthermore, *Ilp6* expression was reduced significantly in middle-aged males after sleep fragmentation (*P* < 0.01; **Figure 5A**).

Since, Insulin signaling may be neuroprotective, the higher IPC *Ilp* levels in the neurons of middle-aged flies could be an attempt at protection against neurotoxic byproducts, which they are unable to remove as efficiently as neurons in younger flies. To test this theory we examined the transcript levels of the *Drosophila Insulin-like receptor* (*InR*) in the head. After sleep fragmentation *InR* levels were significantly higher in both young (*P* < 0.05) and middle-aged (*P* < 0.01) males (**Figure 5B**). However, in young males *InR* levels were back to normal after 4 days of recovery, while in middle-aged males *InR* expression was still significantly elevated (*P* < 0.01; **Figure 5B**). The increased levels of InR expression in the head of middle aged flies could indicate that insulin is induced as a neural protective mechanism, similar to *cnc* and the UPR genes.

## Discussion

Our data demonstrates that sleep fragmentation induces multiple neuroprotective systems, most likely to counteract neuronal burden because of increased oxidative stress and/or cytotoxic metabolites. The fact that neuroprotective pathways remain activated in older flies beyond sleep fragmentation suggests that it might represent a strong stressor for the brain during later life.

Poor sleep quantity or quality produces ROS accumulation in neurons, inducing oxidative stress, leading to cellular damage (Nikonova et al., 2010). As organisms age their ability to control oxidative stress weakens, but it is not clear how diminished sleep quality (as opposed to quantity) influences the expression of antioxidative systems or how age affects the ability of cells to induce these systems. Our study demonstrates that recurrent sleep fragmentation is sufficient to induce expression of the ARE transcription factor *cap-n-collar* (*cnc*), the *Drosophila NFE2L2* homolog, in both young and middle-aged fly brains. Furthermore, in both age groups the expression of *cnc* was still significantly upregulated after 4 days of sleep recovery (see **Figure 3A**). Thus, recurrent sleep fragmentation increases in neuronal oxidative stress may remain even after normal sleep is re-established. On the other hand, neurons may maintain this system in an elevated state in anticipation of future sleep disruptions.

Age-related diseases, including Alzheimer’s and Parkinson’s, are characterized by accumulation and aggregation of misfolded proteins, indicating a decline in molecular chaperone systems. One such system, the ER stress response, also known as the UPR, is responsible for maintaining ER protein homeostasis. We show that under normal sleep conditions older flies express these ER molecular chaperones at significantly lower levels than younger flies. We also show that sleep fragmentation induces ER molecular chaperone proteins in young and middle-aged flies. However, in older flies these genes are still upregulated after 4 days of recovery sleep. This indicates that middle-aged flies are not able to recover from sleep fragmentation as easily as young flies. This may also indicate that older flies have an impaired UPR that is not able to bring them back to a homeostatic level where they can consolidate sleep. In mice and flies, similar to what we demonstrate with sleep fragmentation in flies, prolonged wakefulness or sleep deprivation was shown to activate the UPR (Shaw et al., 2000; Naidoo et al., 2005). Interestingly, it was reported that UPR can influence recovery sleep following sleep loss and that inducing high-levels of ER stress in young flies fragments baseline sleep and alters recovery sleep, demonstrating a direct link between ER stress and sleep (Brown et al., 2014). In fact, flies having a mutation in the *Drosophila* UPR homolog *Calreticulin* (*Crc*), which in our study was significantly upregulated by sleep fragmentation, sleep significantly less than controls (Harbison and Sehgal, 2008).

There is evidence that upregulation of the ER molecular chaperon complex during sleep fragmentation controls the signaling of dopaminergic neurons. Interestingly, when murine dopaminergic neurons are put under stress they upregulate the *Crc* homolog *Calreticulin* (*Calr*; Lee et al., 2003). Furthermore, rat brains exposed to neurotoxic 6-hydroxydopamine induce the expression of not only *Calr*, but also *Pdia3* (*Drosophila ERp60*; Lessner et al., 2010). This leads to the possibility that the ER molecular chaperone system is neuroprotective, actively protecting dopaminergic signaling in adults. On the other hand, a continual increase in the production and reuptake of dopamine may result in dopamine leakage into the cytosol, which can lead to neuronal cell death (Chen et al., 2008). Furthermore, dopaminergic signaling is implicated in wake promotion and elevated dopamine levels may themselves disturb normal sleep patterns (Gruner et al., 2009). In our system, sleep fragmentation was sufficient to significantly increase the expression of genes involved in regulating dopamine production, secretion and re-uptake, indicating a significant increase in dopaminergic signaling. Furthermore, in middle-aged flies 4 days of sleep recovery was not sufficient to bring these elevated levels down to normal. Whereas short-term sleep fragmentation may induce dopaminergic neuroprotective ER chaperones, this same system, if continually induced, may lead to dopamine neurotoxicity. Many Parkinson’s disease patients complain of disrupted or poor sleep, which in the long-term could explain the reduction in dopamine signaling (Swick, 2012). Sleep fragmentation in the elderly might exacerbate Parkinson’s disease, where continual sleep fragmentation would damage dopaminergic neurons.

In our study we compared the effects of recurrent sleep fragmentation on insulin system genes. In the brains of older flies sleep fragmentation induces the expression of both insulin-like peptides and the insulin receptor, and this induction is maintained even after 4 days of recovery sleep. In young flies, sleep fragmentation had no effect on insulin-like peptide expression and only a transient increase in insulin receptor expression. Previously, it was demonstrated that insulin signaling is conducive to sleep consolidation in elderly *Drosophila* (Cong et al., 2015), on the other hand, lowering systemic insulin signaling in *Drosophila* adults reduced age-related increases in sleep fragmentation (Metaxakis et al., 2014). Therefore, how beneficial insulin signaling is as a neuroprotective system for neuronal damage induced by age-related sleep fragmentation is still under debate. Emerging evidence suggests that mitochondria are an insulin-sensitive source of ROS essential for insulin receptor activation in neurons (Rajala and Anderson, 2001). Some evidence suggests that insulin-stimulated H_2_O_2_ plays a critical role in early insulin receptor signaling in neuronal cells (Ramalingam and Kim, 2015). Aging is accompanied by a decline in brain mitochondrial functions, including respiration with the complex I substrate NADH, enzymatic activity of complex I and complex IV, and ATP production (Ferguson et al., 2005; Jones and Brewer, 2010). Therefore, the age-related decline in mitochondrial function seems to disrupt insulin receptor activation in neurons and lead to the development of cerebral insulin resistance in old age. In view of the ultrasensitivity of insulin receptor autophosphorylation to antioxidant activity in neurons, the elevated activity of the antioxidant systems in Parkinson’s disease and Alzheimer’s disorder may contribute to dysfunctional insulin receptor activation and central insulin resistance, which would lead to a decline in synapses and synaptic function (Freiherr et al., 2013).

It could be argued that a caveat of our findings is that we obtained them using the non-mammalian model system *Drosophila melanogaster*. However, it is well established that the characteristics of *Drosophila* sleep are similar to mammalian sleep (Hendricks et al., 2000; Shaw et al., 2000). *Drosophila* sleep is associated with changes in brain activity and shows circadian regulation (Shaw et al., 2002). Furthermore, similar to mammals, in *Drosophila* increases in sleep duration after sleep deprivation are relative to the amount of deprivation (Huber et al., 2004). Furthermore, sleep deprivation disrupts learning and memory consolidation in *Drosophila* (Huber et al., 2004; Li et al., 2009; Seugnet et al., 2009; Le Glou et al., 2012). Moreover, similar to humans, as flies age sleep becomes more fragmented (Koh et al., 2006). Finally, similar to humans, fruit flies are diurnal. All of this demonstrates important similarities between *Drosophila* and mammalian sleep.

We feel we must discuss a few other limitations of our study. In regards to sleep vs. circadian rhythm, given that the sleep/wake-cycle and the control of circadian rhythm are highly intertwined biological processes (Naylor et al., 2000; Cedernaes et al., 2015), our study cannot discriminate whether the observed molecular effects are mainly driven by sleep fragmentation (without changing total sleep duration over 24 h), circadian disruption, or both. Furthermore, unlike other *Drosophila* studies, we chose to use light intervention, instead of mechanical intervention, to disrupt the night time sleep pattern. Our thinking was that although mechanical intervention certainly causes sleep fragmentation (and intertwined circadian disruption), it may also cause physical stress in flies. In other words, we would not be able to discriminate whether the observed molecular effects were driven by sleep fragmentation (and interlinked circadian disruption), physical stress, or both. We felt that light intervention would put less physical stress on the flies. In future we believe it would be beneficial to test this idea by performing a side-by-side experiment comparing mechanical with light intervention for inducing sleep disruption. Finally, it must be mentioned that male and female flies do not share exactly the same sleep patterns (Ho and Sehgal, 2005), therefore to truly understand the effects of sleep fragmentation on genes regulating neuronal protection, these studies should be repeated using female flies. Also, we only used one strain of *Drosophila*, which one could argue we are looking at a specific genomic effect that may vary between strains.

## Conclusion

Our study demonstrates that neurons in young and old male flies are both affected by recurrent sleep fragmentation, yet, older neuronal systems may struggle to regain cellular homeostasis disrupted by continual poor sleep quality. In fact, the three systems we studied, insulin, antioxidative and the UPR, may interact to protect neurons against oxidative stress and cytotoxic metabolites. When sleep quality is poor, ROS build up in neurons, this would induce ARE genes in an attempt to reduce neuronal ROS levels. Increases in ROS might damage mitochondria, leading to decreased levels of ATP, which would induce the ER UPR response. At the same time, increased ROS levels would aid in neuronal insulin signaling, which would inhibit cytochrome c release, necessary to protect neurons from UPR induced apoptosis. When we are young, and impaired sleep quality is intermittent, these systems would interact to protect neurons from damage and help to restore normal sleep patterns. As we age there is a decrease in sleep quality, which could lead to increases in oxidative stress and cytotoxic metabolites. At the same time basal expression levels of the systems required to combat these stresses are reduced. Continual elevated levels in cellular ROS in older individuals, due to increased sleep fragmentation, might eventually lead to neuronal insulin resistance. Furthermore, constant activation of the UPR, with a concurrent reduction in insulin signaling, would induce autophagy, leading to programmed cell death. Clearly, it is important to understand how these cytoprotective systems react not only to sleep quality, but also how age affects the efficiency of these systems.

## Author Contributions

MW: Designed project; designed, carried out and analyzed experiments, wrote manuscript, Provided funding. EP, ME, JC, DE, LL, TM, and EP: Designed, carried out and analyzed experiments. RF, CB, and HS: Designed project, wrote manuscript, and provided funding.

## Conflict of Interest Statement

The authors declare that the research was conducted in the absence of any commercial or financial relationships that could be construed as a potential conflict of interest.

## Footnotes

1 http://flystocks.bio.indiana.edu/
